# Association of genetic variants with extravascular complications and cytokine production in Takayasu arteritis: a cross-sectional study

**DOI:** 10.1093/rap/rkaf133

**Published:** 2025-12-03

**Authors:** Farzana Shumy, Kotaro Matsumoto, Masaru Takeshita, Hiroya Tamai, Keiko Yoshimoto, Mayu Magi, Hiroto Yoshida, Yuko Kaneko

**Affiliations:** Division of Rheumatology, Department of Internal Medicine, Keio University School of Medicine, Tokyo, Japan; Division of Rheumatology, Department of Internal Medicine, Keio University School of Medicine, Tokyo, Japan; Division of Rheumatology, Department of Internal Medicine, Keio University School of Medicine, Tokyo, Japan; Division of Rheumatology, Department of Internal Medicine, Keio University School of Medicine, Tokyo, Japan; Division of Rheumatology, Department of Internal Medicine, Keio University School of Medicine, Tokyo, Japan; Product Research Department, Chugai Pharmaceutical Co. Ltd, Kanagawa, Japan; Product Research Department, Chugai Pharmaceutical Co. Ltd, Kanagawa, Japan; Division of Rheumatology, Department of Internal Medicine, Keio University School of Medicine, Tokyo, Japan

**Keywords:** Takayasu arteritis, extravascular complications, genetic associations

## Abstract

**Objective:**

Genetic variants in the chromosome 21q22 (chr21q22) region are shared among patients with Takayasu arteritis (TAK), inflammatory bowel disease (IBD) and spondyloarthritis (SpA), contributing to specific macrophage inflammation. This study aims to clarify the impact of this region on the clinical and molecular phenotypes of TAK.

**Methods:**

In this cross-sectional study, 71 TAK patients from Keio University Hospital were included. Single-nucleotide polymorphisms (SNPs) in the chr21q22 region were identified through genomic DNA sequencing in 25 patients and serum proteome analysis was conducted in 17 patients. Interleukin-17C (IL-17C)-induced cytokine production was measured via whole blood cytometry by time of flight (CyTOF) in five patients with chr21q22 SNP accumulation and five without.

**Results:**

Among the 71 patients, 11% had IBD and 8.5% had SpA. Complications of IBD and SpA were strongly associated with chr21q22 SNP accumulation such as rs2242944, rs9808651 and rs2836882. Serum proteomic analysis revealed significantly elevated levels of IL-17C in TAK patients with chr21q22 SNP accumulation. IL-17C stimulation of whole blood from patients with chr21q22 SNP accumulation resulted in increased IL-6- and TNF-α-producing cells compared with those without SNP accumulation.

**Conclusion:**

SNPs in the chr21q22 region and elevated IL-17C levels may contribute to the pathophysiology of TAK–IBD–SpA comorbidity. These insights advance our understanding of the genetic and inflammatory mechanisms underlying extravascular complications in TAK.

Key messages

- In our TAK cohort, SNP accumulation in the chr21q22 region was significantly associated with comorbidities of IBD and SpA.
- Serum IL-17C level is elevated in the serum of TAK patients with chr21q22 SNP accumulation and is associated with cytokine production from monocytes.
- SNPs in the chr21q22 region and elevated IL-17C levels may contribute to the molecular pathogenesis of TAK–IBD–SpA comorbidity.

## Introduction

The genetic predisposition to Takayasu arteritis (TAK) has primarily been analysed in relation to the human leucocyte antigen (HLA) region [1]. HLA-B52:01 is an established susceptibility locus for TAK [2], while recent findings have identified HLA-G as an additional associated locus [3]. Furthermore, HLA-B67:01 has been confirmed as a novel susceptibility allele for TAK in the Japanese population [1]. The HLA-B52:01 allele has also been reported to influence disease phenotype, with patients carrying this allele being more likely to develop extensive aortic involvement and requiring higher doses of glucocorticoids to manage increased disease activity [4, 5]. Another report found an association between HLA-B52:01 and the high prevalence of inflammatory bowel disease (IBD) as a comorbidity in TAK patients [1, 6]. HLA-B27 negativities have been reported to be associated with a high prevalence of spondyloarthritis (SpA) as a comorbidity in TAK patients [7, 8]. However, the influence of the non-HLA region on disease phenotype in TAK is less well documented compared with that of the HLA region.

Non-HLA regions, such as CFL2, DUSP22, FCGR3A, HSPA6, IL-12B, KLHL-33, LILRA3, PTK2B, PTPN2, SVEP1 and VPS8, have been reported to be associated with TAK [9]. A recent report, primarily authored by researchers focusing on IBD, identified an intergenic haplotype on chromosome 21q22 (chr21q22), rs2836882, that is shared among patients with IBD, SpA and TAK [9, 10], indicating that the chr21q22 region may be associated with extravascular complications of TAK. However, this finding has not been sufficiently analysed from the perspective of TAK, leaving its relevance in TAK disease mechanisms unclear. It also remains unclear how the accumulation of single-nucleotide polymorphisms (SNPs) in chr21q22 affects the disease phenotype of TAK.

Extravascular complications in TAK are less commonly documented compared with vascular features. IBD is observed in 6.3–9.3% of cases [11]. Regarding the overall prevalence of TAK with SpA, individual studies report varying rates, with Güzel Esen *et al.* reporting the highest at 20.3%, while others range between 5.7% and 8% [12–14]. Eye involvement, such as uveitis, and skin manifestations, such as erythema nodosum, have also been reported [7]. The primary objective of this study is to validate the impact of gene polymorphisms in the chr21q22 region on the clinical characteristics of extravascular complications in TAK. The secondary objective is to elucidate the immunological role of these polymorphisms in TAK.

## Methods

### Patients

This study employed a cross-sectional design, including a total of 71 consecutive patients who attended Keio University Hospital between January 2012 and December 2023. All eligible patients during this period who met the 2022 ACR/EULAR classification criteria [15] were included. A comprehensive chart review was performed to assess the timing and clinical characteristics of extravascular complications involving the intestines, joints, eyes and skin. The diagnosis of IBD was based on the Japan College of Gastroenterology guideline [16]. The diagnosis of SpA was established based on the Assessment of SpondyloArthritis international Society classification criteria [17]. The study was approved by the Keio University Ethics Committee (approval number 20140479, 23 March 2015) and was in accordance with the Declaration of Helsinki.

Serum and whole blood samples were collected from patients who provided consent to investigate the association between extravascular complications and SNPs in patients with TAK. Peripheral blood samples for genomic analysis were obtained from 25 patients who provided consent. Among these, serum samples at the time of newly diagnosed or relapsed disease were available in 17 patients, in whom proteomic assays were performed. Of these 17 patients, 5 showed SNP accumulation in the chr21q22 region and serum samples were available for 4 of them. For cytometry by time of flight (CyTOF) analysis, these five patients with SNP accumulation were compared with five patients without accumulation.

### Genomic DNA sequencing

Genomic DNA was extracted from whole blood samples of 25 TAK patients using the DNA Purification Kit (Maxwell, Tokyo, Japan). To identify SNPs in the chr21q22 region, a 2518-bp segment spanning positions 21:39092400 to 21:39094917 was amplified using a specific primer pair: a forward primer (CCTTTTAATTGATCTGTATCTCCTATG) and a reverse primer (CTGTTCTTCAGCTTTCCATGTC), based on the previous study [10]. The amplification process involved 40 cycles to generate sufficient copies of the target DNA region for sequencing. The amplified DNA was subjected to agarose gel electrophoresis to confirm successful amplification and verify the expected fragment size. DNA bands corresponding to the target fragment were excised from the gel and purified using the Gel/PCR Purification Kit (Favorgen, Ping Tung, Taiwan) to ensure removal of contaminants and preparation of high-purity DNA for sequencing.

The 2518-bp segment contained variations at the following positions: 21:39092998 (rs4817983), 21:39093140 (rs4817984), 21:39093252 (rs2242944), 21:39093586 (rs4817986), 21:39093608 (rs2836878), 21:39093975 (rs4817987), 21:39094373 (rs2836881), 21:39094542 (rs9808651), 21:39094644 (rs2836882) and 21:39094818 (rs2836883). To achieve comprehensive coverage and accuracy, multiple primers were utilized during sequencing. Forward primers included ACAAAAATTAGCCAGGCGTG and GAGGGAGAACGGACCC, while reverse primers included CTGTTCTTCAGCTTTCCATGTC and TAGACTTGCTGCTAGCTCTTG.

The presence of homozygous and heterozygous variants was determined by inspecting the sequence alignment at the target SNP positions. Homozygous sites showed a single peak for each nucleotide while heterozygous sites displayed overlapping peaks. The SNP score was calculated by assigning 1 point for homozygotes and 0.5 points for heterozygotes, based on the previous study [18].

### Serum proteomic analysis

Serum proteomic analysis was performed on 17 newly diagnosed or relapsed TAK patients using four targeted Olink proteomics panels: inflammation, immune response, cardiovascular II and cardiovascular III. Biomarker detection thresholds were determined based on the mean values from triplicate negative controls for each run. The samples were classified into two groups, those with and without chr21q22 accumulation, according to the results of genomic DNA sequencing. Comparative analysis was performed between the two groups. A fold change (FC) cut-off of 1.5 was applied to identify significant differences in biomarker levels.

### CyTOF

Cytokine production response to IL-17C stimulation was evaluated using CyTOF in whole blood samples from five TAK patients with chr21q22 SNP accumulation and five without. Recombinant IL-17C (catalogue no. 1234-IL-025, R&D Systems, Minneapolis, MN, USA) was added to whole blood samples at a final concentration of 50 ng/ml. To facilitate intracellular cytokine detection, a protein transport inhibitor cocktail (catalogue no. 00-4980-93, Thermo Fisher Scientific, Waltham, MA, USA) was added simultaneously and the samples were incubated for 4 h. Afterward, intracellular staining was performed using the Transcription Factor Staining Buffer Set (catalogue no. 00-5523-00, Thermo Fisher Scientific). The antibodies used in the analysis are detailed in Supplementary Table S1, available at *Rheumatology Advances in Practice* online. The data were analysed using FlowJo software (BD Biosciences, San Jose, CA, USA) to quantify cytokine production and immune cell profiles and to enable advanced visualization.

### Statistical analysis

Statistical analyses were conducted using R version 4.1.3 (R Foundation for Statistical Computing, Vienna, Austria) and Python version 3.10 (https://www.python.org/downloads/release/python-3100/). For the analysis of the association between extravascular complications and chr21q22 SNP accumulation, categorical variables were compared using chi-squared tests and continuous variables such as SNP scores were analysed using the Mann–Whitney U test. In the analysis of proteomic data, biomarker levels were compared using two-sided *t*-tests. *P*-values <0.05 were considered statistically significant in all analyses. Due to the small sample size, correction for multiple comparisons was not applied.

## Results

### Prevalence and spectrum of extravascular complications in TAK

Among the 71 TAK patients, 22.5% (16/71) had extravascular complications while the remaining 55 patients did not. The median age at TAK diagnosis was 42 years [interquartile range (IQR) 25–54] in patients with extravascular complications and 32 years (IQR 25–54) in those without (*P* = 0.78). The proportions of female patients were 75% (12/16) and 76% (42/55), respectively. All patients were of Asian descent. These findings indicate no significant differences in age, sex or ethnicity between the two groups.

**Figure 1.**
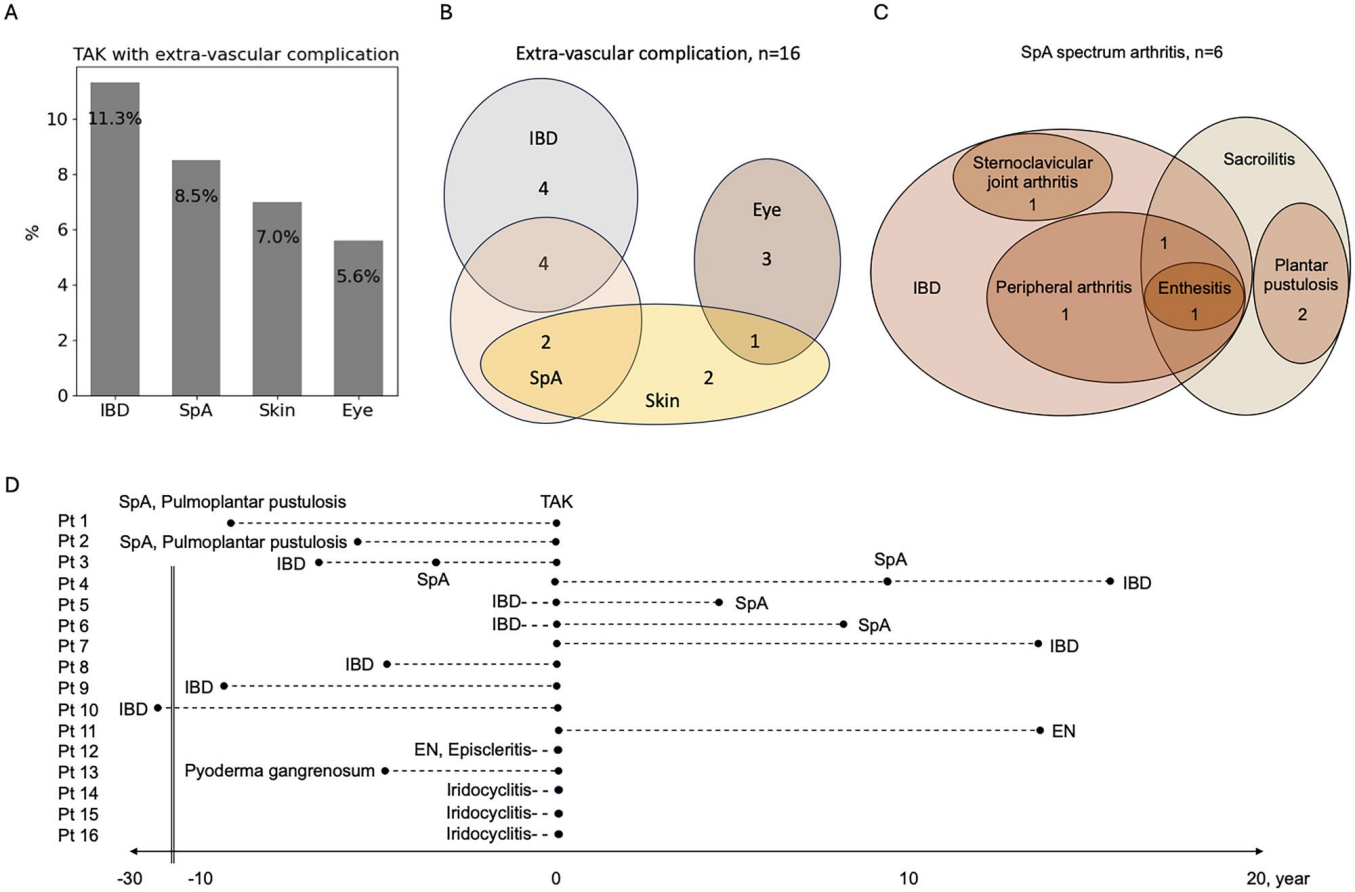
Timing and clinical characteristics of extravascular complications in TAK. **(A)** The proportion of TAK patients exhibiting extravascular complications. **(B)** Detailed distribution of extravascular complications in TAK patients. **(C)** Detailed manifestations of SpA spectrum disorder. **(D)** Timing of the onset of extravascular complications in TAK patients. EN: erythema nodosum

The specific complications included IBD in 11.3% (8/71), SpA spectrum arthritis in 8.5% (6/71), skin involvement in 7.0% (5/71) and eye involvement in 5.6% (4/71) (Fig. 1A). A Venn diagram highlights overlaps among extravascular complications (Fig. 1B). Eight patients had IBD, comprising six with ulcerative colitis and two with Crohn’s disease. Among these, four also had SpA. Of the six patients with SpA, four were associated with IBD, presenting with sacroiliitis, peripheral arthritis, enthesitis and sternoclavicular joint arthritis. The remaining two had SpA characterized by sacroiliitis and plantar pustulosis (Fig. 1C).

Skin manifestations were present in five patients, with two showing plantar pustulosis, two showing erythema nodosum and one pyoderma gangrenosum. Eye complications were observed in four patients, including three with iridocyclitis and one with episcleritis.

The timing of the onset of extravascular complications is summarized in Fig. 1D. Half of the SpA cases (3/6) developed before TAK onset, while the other half (3/6) occurred afterward. In contrast, 75% (6/8) of IBD cases preceded TAK, while 25% (2/8) developed afterward. All four patients with eye complications experienced them concurrently with TAK.

### Extravascular complications associated with chr21q22 SNP accumulation

**Figure 2.**
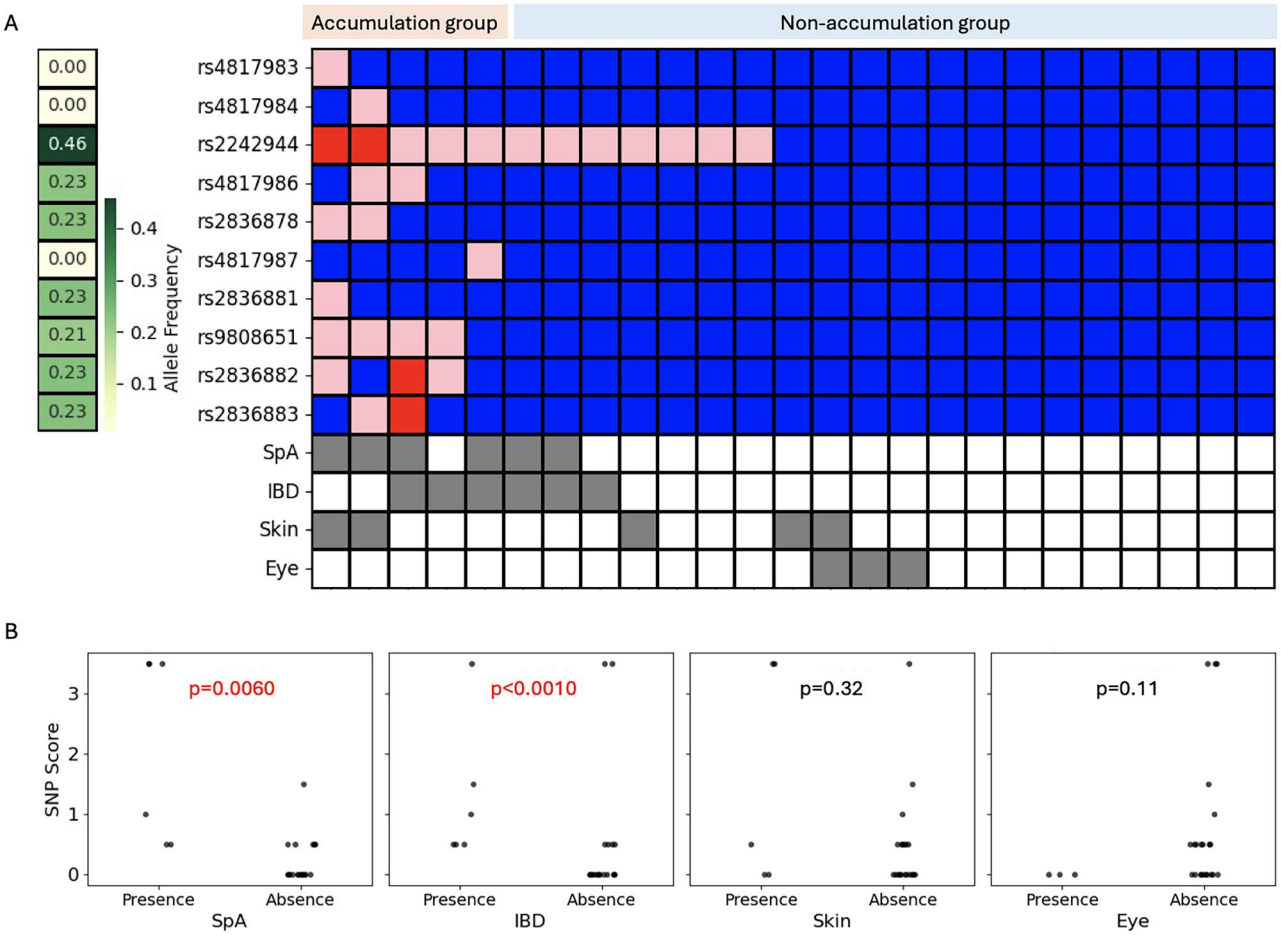
Association between chr21q22 SNPs and extravascular complications in TAK. **(A)** The association between the presence of SNPs in the chr21q22 region and extravascular involvement. Red: homozygous variant; pink: heterozygous variant; blue: wild type. Presence (grey) and absence (white) of each extravascular complication. **(B)** The association between the SNP score in chr21q22 and the presence of each extravascular complication

We identified SNPs in the chr21q22 region, a 2518-bp segment spanning positions 21:39092400 to 21:39094917. The presence of each SNP is shown, along with the allele frequency for each SNP in the Japanese population, as well as the presence of SpA, IBD and skin and eye involvement in each patient (Fig. 2A).

The SNP scores were calculated for each patient and were compared based on the presence or absence of each extravascular complication. Higher scores were associated with the accumulation of SNPs in chr21q22. TAK patients with IBD [0.75 (IQR 0.5–2) *vs* 0 (0–0.5), *P* = 0.0060] and SpA [2.25 (IQR 0.5–3.5) *vs* 0 (0–0.5), *P* < 0.0010] showed significantly higher SNP scores, while no significant differences were observed for skin [0.5 (IQR 0–3.5) *vs* 0 (0–0.5), *P* = 0.32] and eye involvement [0 (IQR 0–0) *vs* 0.5 (0–0.625), *P* = 0.11].

Regarding individual alleles, rs2242944 (100% *vs* 24%; *P* < 0.0010), rs9808651 (50% *vs* 0%; *P* = 0.0010), rs2836882 (38% *vs* 0%; *P* = 0.053) were significantly more common in TAK patients with IBD or SpA compared with those without these complications.

### Serum IL-17C is upregulated in TAK patients with chr21q22 SNP accumulation

**Figure 3.**
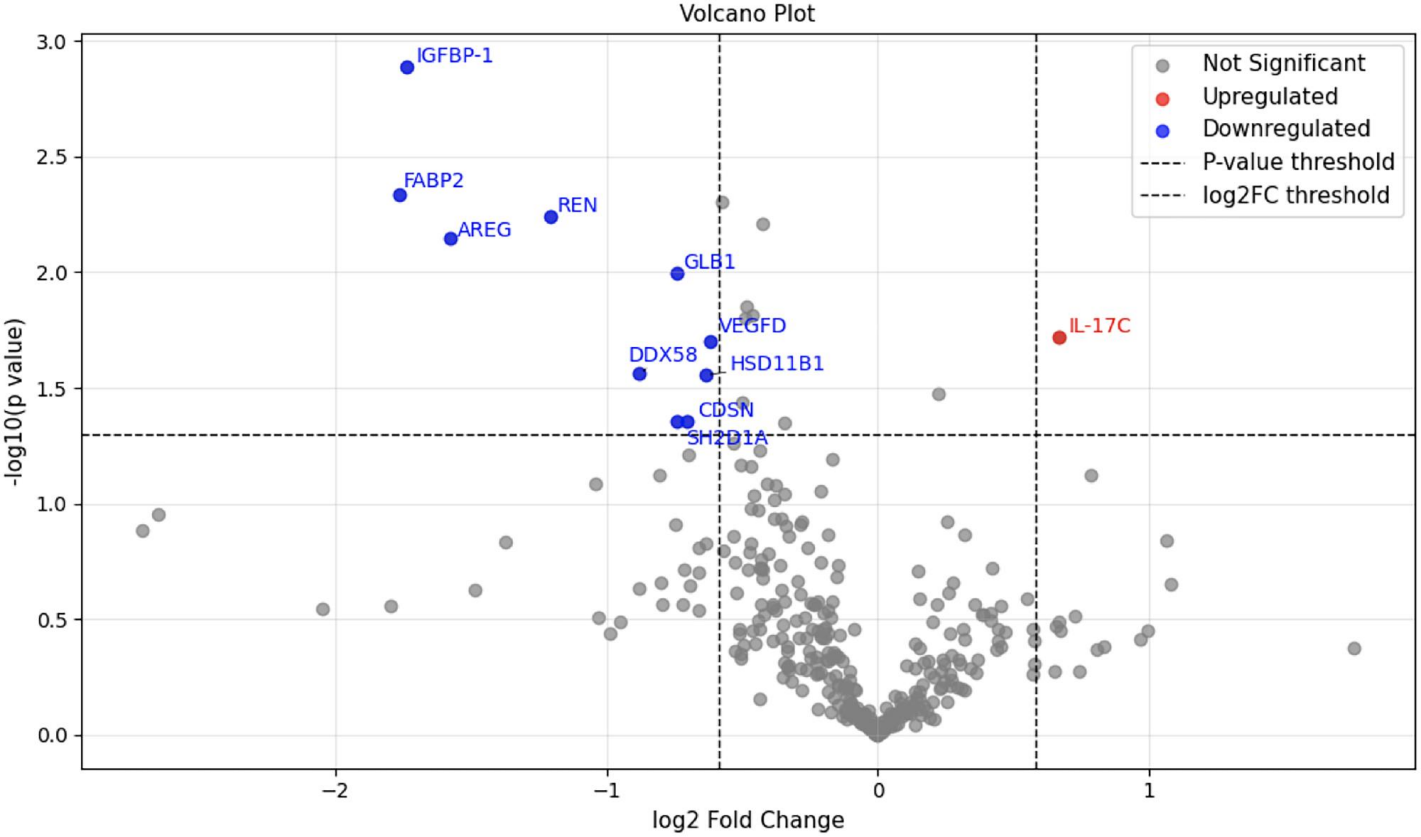
Results of serum proteomic analysis in TAK patients with and without chr21q22 SNP accumulation. Volcano plot comparing the results of serum proteomic analysis between TAK patients with and without chr21q22 SNP accumulation. Red dot: upregulated proteins in TAK patients with chr21q22 accumulation group; blue dot: downregulated proteins in the same group; grey dot: not significant proteins

Since SNPs including rs2836882 are associated with inflammatory receptors and cytokine production [10], we investigated serum proteomic analysis and its association with SNPs in the chr21q22 region. For the analysis, patients were categorized into those with two or more SNPs (accumulation group) and those with one or no SNPs (non-accumulation group). To investigate molecular differences between TAK patients with and without chr21q22 SNP accumulation, serum protein expression levels were compared between the accumulation and non-accumulation groups. The clinical characteristics of the patients used in the analysis are shown in Table 1. Serum proteomic analysis revealed significantly elevated levels of IL-17C in the accumulation group (FC = 1.6, *P* = 0.019) (Fig. 3). Conversely, significant decreases were noted in the expression of AREG, DDX58, FABP2, GLB1, IGFBP-1, REN and SH2D1A in accumulation group.

**Table 1:** Clinical characteristics of TAK patients used in Olink proteomic analysis.

Characteristics	Accumulation group (*n* = 4)	Non-accumulation group (*n* = 13)	*P*-value
Age, years, median (IQR)	47 (29–58)	41 (26–47)	0.43
Female, *n* (%)	3 (75)	6 (46)	0.30
Newly diagnosed, *n* (%)	3 (75)	9 (69)	0.82
Comorbidities, *n* (%)			
IBD	3 (75)	0 (0)	**<0.0010**
SpA	3 (75)	0 (0)	**<0.0010**
Skin	1 (25)	0 (0)	0.078
Eye	0 (0)	1 (7.7)	0.46
Laboratory tests			
CRP, mg/dl, median (IQR)	2.5 (1.0–18)	1.0 (0.1–5.2)	0.46
Immunological treatments, *n* (%)			
Prednisolone	1 (25)	4 (31)	0.82
Tocilizumab	1 (25)	0 (0)	0.078
Golimumab	1 (25)	0 (0)	0.078
Azathioprine	0 (0)	2 (15)	0.28

Significant *P*-values in bold.

### Cytokine production response to IL-17C upregulated in TAK patients with chr21q22 SNP accumulation

IL-17C is secreted by epithelial cells and keratinocytes and it has been reported that stimulation of monocytic cell lines by IL-17C induces inflammatory cytokines [19, 20]. Therefore, to investigate the role of IL-17C on the chr21q22 region, cytokine production response was compared using whole blood samples from TAK patients with and without chr21q22 SNP accumulation. The clinical characteristics of the patients used in the analysis are shown in Table 2.

**Table 2:** Clinical characteristics of TAK patients used in CyTOF analysis.

Characteristics	Accumulation group (*n* = 5)	Non-accumulation group (*n* = 5)	*P*-value
Age, years, median (IQR)	53 (44–68)	45 (32–51)	0.14
Female, *n* (%)	4 (80)	2 (40)	0.19
Newly diagnosed, *n* (%)	0 (0)	0 (0)	–
Comorbidities, *n* (%)			
IBD	3 (60)	0 (0)	**0.019**
SpA	4 (80)	0 (0)	**0.0036**
Skin	2 (40)	1 (20)	0.49
Eye	0 (0)	0 (0)	–
Laboratory tests			
CRP, mg/dl, median (IQR)	0.02 (0.02–0.14)	0.04 (0.01–0.2)	0.90
Immunological treatments, *n* (%)			
Prednisolone	5 (100)	5 (100)	–
Tocilizumab	2 (40)	3 (60)	0.53
Infliximab	1 (20)	1 (20)	1.0
Adalimumab	1 (20)	0 (0)	0.22
Methotrexate	2 (40)	1 (20)	0.49
Azathioprine	1 (20)	0 (0)	0.22

Significant *P*-values in bold.

The t-distributed stochastic neighbour embedding (t-SNE) map, overlaying cell surface markers with intracellular markers, is shown in Supplementary Fig. S1, available at *Rheumatology Advances in Practice* online. This analysis revealed that cytokine production in response to IL-17C stimulation was predominantly concentrated in CD14^+^ monocytes.

**Figure 4.**
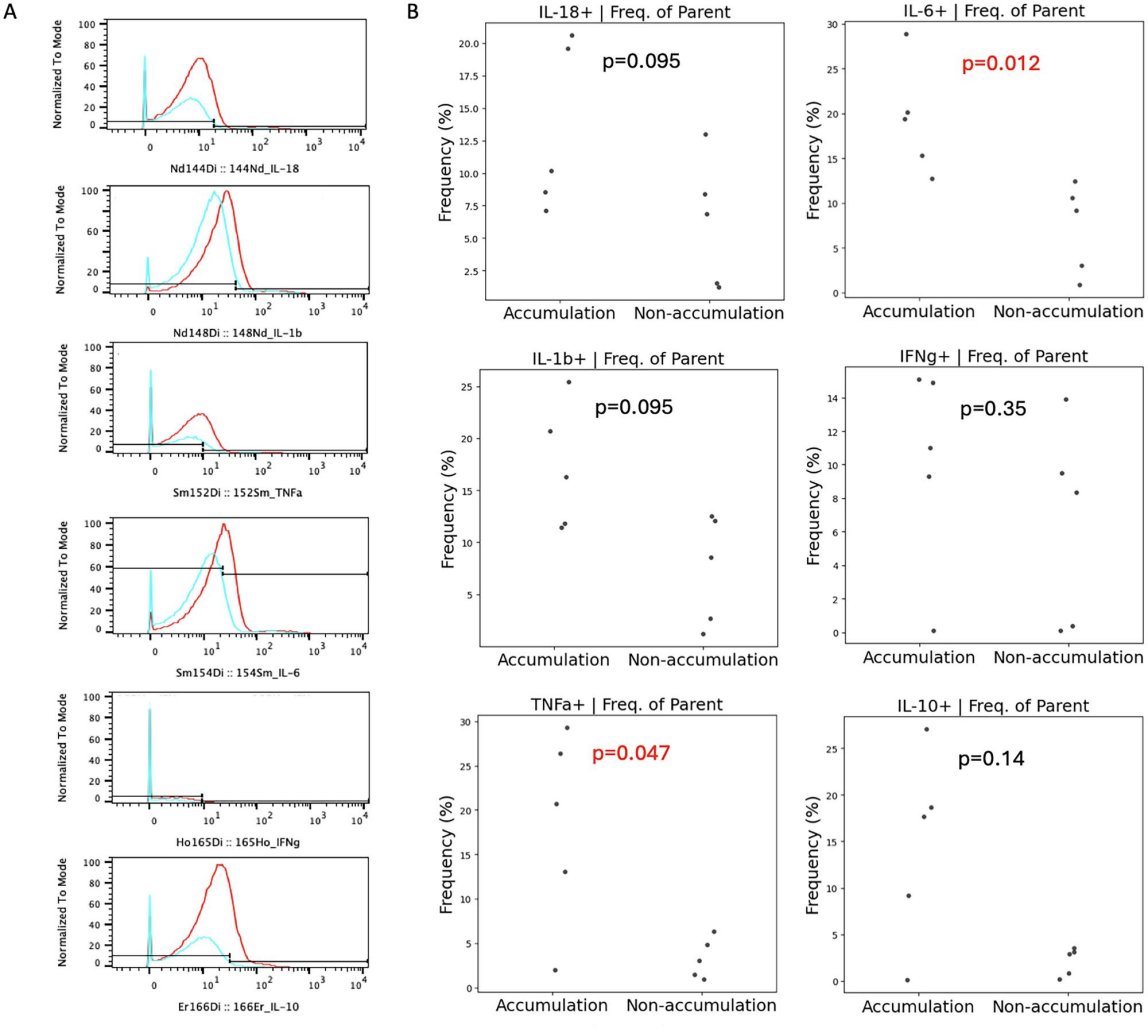
Results of CyTOF analysis in TAK patients with and without chr21q22 SNP accumulation. **(A)** Representative intracellular cytokine production in monocytes before (blue) and after (red) IL-17C stimulation assessed by CyTOF. **(B)** Comparison of intracellular cytokine-producing monocytes stimulated with IL-17C between TAK patients with and without chr21q22 SNP accumulation

Fraction of singlet/live/CD45^+^/CD14^+^ cells were gated. A representative histogram of cytokine-producing cells following IL-17C stimulation is shown in Fig. 4A. The proportion of cytokine production induced by stimulation was calculated using the unstimulated condition as a reference. IL-17C stimulation resulted in a significant increase in TNF-α (20.7% *vs* 3.0%; *P* = 0.047) and IL-6 (19.4% *vs* 9.1%; *P* = 0.012) production in monocytes from patients with chr21q22 SNP accumulation. However, no significant changes were observed in IL-1β, IL-10, IL-18 or IFN-γ levels (Fig. 4B). These findings suggest that IL-17C may enhance pro-inflammatory cytokine production from monocytes in the context of chr21q22 SNP accumulation, potentially contributing to disease pathology.

## Discussion

This study provides new insights into the prevalence and spectrum of extravascular complications in TAK patients and their association with an intergenic haplotype on chr21q22. We identified a subset of TAK patients with IBD and SpA who exhibited significant SNP accumulation in chr21q22, elevated serum IL-17C levels and enhanced IL-6 and TNF-α production by monocytes.

Our findings extend prior genetic studies in TAK, which have predominantly focused on HLA loci [1–6]. While these HLA associations explain part of TAK’s susceptibility and phenotypic variation, the role of the non-HLA region remains unexplored. The chr21q22 region, recently implicated in IBD and SpA [10], has not been thoroughly studied in TAK. Our data suggest that this intergenic region may represent a novel susceptibility locus in TAK patients with extravascular manifestations.

Approximately 22.5% of TAK patients in our cohort presented with extravascular complications, with IBD and SpA being the most common, in line with previous studies [11–14]. Notably, SNPs such as rs224944, rs9808651 and rs2836882 were significantly enriched in TAK patients with IBD and SpA, suggesting a genetic overlap between TAK and these inflammatory conditions and supporting the concept of a shared immunogenetic background [6, 10, 21].

Serum proteomic analysis, conducted to investigate potential immunopathogenetic mechanisms, revealed significant elevation of IL-17C in patients with chr21q22 SNP accumulation. Unlike IL-17A/F, IL-17C acts through the IL-17RA/IL-17RE heterodimeric receptor complex [22]. Binding of IL-17C to IL-17RE (high affinity) and IL-17RA (low affinity) induces pro-inflammatory cytokines such as TNF-α and IL-1β [19, 20]. IL-17C has been implicated in inflammatory diseases affecting the intestines and skin. In IBD, it contributes to pathogenesis by forming an inflammatory amplification loop between epithelial cells and Th17 cells [23]. Elevated IL-17C expression has been reported in psoriasis, hidradenitis suppurativa and atopic dermatitis [19, 24]. In our TAK cohort, elevated IL-17C may reflect enhanced intestinal and cutaneous inflammation. Together with our *in vitro* data showing that IL-17C stimulation augments IL-6 and TNF-α production by monocytes, these findings suggest that IL-17C derived from intestinal and skin lesions could contribute to systemic inflammation and disease activity in TAK.

Monocytes and macrophages play a key role in the pathogenesis of TAK [25, 26]. A previous report showed that an intergenic haplotype within the chr21q22 region, drives inflammatory macrophage activation, providing a mechanistic link between TAK, IBD and SpA [10]. Our findings build on this framework by showing that IL-17C, elevated in TAK patients with chr21q22 accumulation, enhances inflammatory cytokine production in CD14^+^ monocytes. This supports the concept that chr21q22-associated genetic variants may potentiate disease through a monocyte-driven inflammatory pathway. Taken together, these data underscore the importance of pathological monocyte/macrophage activation as a therapeutic target in TAK and highlight IL-17C and its receptor complex (IL-17RA/IL-17RE) as potential novel therapeutic targets for genetically defined TAK subsets.

This study has several limitations. First, the small sample size, reflecting the rarity of TAK, limited statistical power and precluded formal sample size calculation, as well as multiple testing correction, increasing the risk of false-positive findings. Second, the cross-sectional design prevents causal inference between chr21q22 SNP accumulation and IL-17C elevation. Third, although clinical data regarding extravascular complications were available for all 71 patients, some laboratory parameters at diagnosis were missing. Fourth, the single-centre, ethnically homogeneous cohort may limit generalizability. Fifth, heterogeneity in disease state, type and timing of extravascular complications and treatment history could have influenced cytokine levels and immune responses. Finally, only a small segment of the chr21q22 region was sequenced, so other relevant variants could have been missed.

Despite these limitations, this is the first study to link chr21q22 variants with extravascular complications in TAK, with consistent diagnosis ensured by strict criteria. The study combined genetic, serum proteomic and functional immune response analyses in the same cohort. Moreover, detailed characterization of extravascular complications, including their prevalence, clinical features and timing, provided a robust clinical context for interpreting genotype–phenotype relationships.

In summary, our study identifies a potential genetically defined subset of TAK patients with chr21q22 SNP accumulation, characterized by elevated IL-17C and increased monocyte inflammatory response. These findings suggest a novel molecular mechanism underlying TAK–IBD–SpA comorbidity and highlight IL-17C as a potential therapeutic target. Larger, multi-ethnic cohorts and functional studies are warranted to validate these associations and explore their clinical implications.

## Supplementary Material

rkaf133_Supplementary_Data

## Data Availability

The raw data supporting the conclusions of this article will be made available by the authors upon reasonable request.

## References

[1] Terao C. Revisited HLA and non-HLA genetics of Takayasu arteritis—where are we? J Hum Genet 2016;61:27–32. 26178430 10.1038/jhg.2015.87

[2] Isohisa I , Numano F, Maezawa H, Sasazuki T. HLA-Bw52 in Takayasu disease. Tissue Antigens 1978;12:246–8. 31708 10.1111/j.1399-0039.1978.tb01332.x

[3] Terao C , Yoshifuji H, Matsumura T et al Genetic determinants, and an epistasis of LILRA3 and HLA-B52 in Takayasu arteritis. Proc Natl Acad Sci USA 2018;115:13045–50. 30498034 10.1073/pnas.1808850115 PMC6304955

[4] Origuchi T , Fukui S, Umeda M et al The severity of Takayasu arteritis is associated with the HLA-B52 allele in Japanese patients. Tohoku J Exp Med 2016;239:67–72. 27193038 10.1620/tjem.239.67

[5] Numano F , Ohta N, Sasazuki T. HLA and clinical manifestations in Takayasu disease. Jpn Circ J 1982;46:184–9. 6120251 10.1253/jcj.46.184

[6] Terao C , Matsumura T, Yoshifuji H et al Takayasu arteritis and ulcerative colitis: high rate of co-occurrence and genetic overlap. Arthritis Rheumatol 2015;67:2226–32. 25931203 10.1002/art.39157

[7] Kwon OC , Lee SW, Park YB et al Extravascular manifestations of Takayasu arteritis: focusing on the features shared with spondyloarthritis. Arthritis Res Ther 2018;20:142. 29996949 10.1186/s13075-018-1643-7 PMC6042334

[8] Mielnik P , Hjelle AM, Nordeide JL. Coexistence of Takayasu’s arteritis and ankylosing spondylitis may not be accidental: is there a need for a new subgroup in the spondyloarthritis family? Mod Rheumatol 2018;28:313–8. 28718336 10.1080/14397595.2017.1341592

[9] Ortiz-Fernández L , Saruhan-Direskeneli G, Alibaz-Oner F et al Identification of susceptibility loci for Takayasu arteritis through a large multi-ancestral genome-wide association study. Am J Hum Genet 2021;108:84–99. 33308445 10.1016/j.ajhg.2020.11.014 PMC7820633

[10] Stankey CT , Bourges C, Haag LM et al A disease-associated gene desert directs macrophage inflammation through ETS2. Nature 2024;630:447–56. 38839969 10.1038/s41586-024-07501-1 PMC11168933

[11] Kilic L , Kalyoncu U, Karadag O et al Inflammatory bowel diseases and Takayasu’s arteritis: coincidence or association? Int J Rheum Dis 2016;19:814–8. 26913584 10.1111/1756-185X.12837

[12] Güzel Esen S , Armagan B, Atas N et al Increased incidence of spondyloarthropathies in patients with Takayasu arteritis: a systematic clinical survey. Joint Bone Spine 2019;86:497–501. 30735804 10.1016/j.jbspin.2019.01.020

[13] Abacar K , Kaymaz-Tahra S, Bayındır Ö et al Frequency and the effects of spondyloarthritis-spectrum disorders on the clinical course and management of Takayasu arteritis: an observational retrospective study. Clin Rheumatol 2024;43:1571–8. 38563865 10.1007/s10067-024-06939-y

[14] Esatoglu SN , Ok AM, Ucar D et al Takayasu’s arteritis: associated inflammatory diseases. Clin Exp Rheumatol 2020;38(Suppl 124):61–8. 31969224

[15] Grayson PC , Ponte C, Suppiah R et al 2022 American College of Rheumatology/EULAR classification criteria for Takayasu arteritis. Ann Rheum Dis 2022;81:1654–60. 36351705 10.1136/ard-2022-223482

[16] Nakase H , Uchino M, Shinzaki S et al Evidence-based clinical practice guidelines for inflammatory bowel disease 2020. J Gastroenterol 2021;56:489–526. 33885977 10.1007/s00535-021-01784-1 PMC8137635

[17] Rudwaleit M , van der Heijde D, Landewé R et al The Assessment of SpondyloArthritis International Society classification criteria for peripheral spondyloarthritis and for spondyloarthritis in general. Ann Rheum Dis 2011;70:25–31. 21109520 10.1136/ard.2010.133645

[18] Kerns SL , Stock RG, Stone NN et al Genome-wide association study identifies a region on chromosome 11q14.3 associated with late rectal bleeding following radiation therapy for prostate cancer. Radiother Oncol 2013;107:372–6. 23719583 10.1016/j.radonc.2013.05.001 PMC3787843

[19] Ramirez-Carrozzi V , Sambandam A, Luis E et al IL-17C regulates the innate immune function of epithelial cells in an autocrine manner. Nat Immunol 2011;12:1159–66. 21993848 10.1038/ni.2156

[20] Li H , Chen J, Huang A et al Cloning and characterization of IL-17B and IL-17C, two new members of the IL-17 cytokine family. Proc Natl Acad Sci USA 2000;97:773–8. 10639155 10.1073/pnas.97.2.773 PMC15406

[21] Pang X , Yang H, Wang C, Tian S. Exploring the causal relationship between Takayasu arteritis and inflammatory bowel disease using Mendelian randomization. Immunol Res 2024;72:707–13. 38536561 10.1007/s12026-024-09476-7

[22] Song X , Zhu S, Shi P et al IL-17RE is the functional receptor for IL-17C and mediates mucosal immunity to infection with intestinal pathogens. Nat Immunol 2011;12:1151–8. 21993849 10.1038/ni.2155

[23] Swedik SM , Madola A, Cruz MA, Llorens-Bonilla BJ, Levine AD. Th17-derived cytokines synergistically enhance IL-17C production by the colonic epithelium. J Immunol 2022;209:1768–77. 36130829 10.4049/jimmunol.2200125 PMC9588696

[24] Navrazhina K , Frew JW, Krueger JG. Interleukin 17C is elevated in lesional tissue of hidradenitis suppurativa. Br J Dermatol 2020;182:1045–7. 31556100 10.1111/bjd.18556 PMC7195726

[25] Watanabe R , Hashimoto M. Pathogenic role of monocytes/macrophages in large vessel vasculitis. Front Immunol 2022;13:859502. 35967455 10.3389/fimmu.2022.859502 PMC9372263

[26] Matsumoto K , Suzuki K, Takeshita M, Takeuchi T, Kaneko Y. Changes in the molecular profiles of large-vessel vasculitis treated with biological disease-modifying anti-rheumatic drugs and Janus kinase inhibitors. Front Immunol 2023;14:1197342. 37197652 10.3389/fimmu.2023.1197342 PMC10183585

