# Supplementary material for: Association of genetic variants with extravascular complications and cytokine production in Takayasu arteritis: a cross-sectional study
Source: Rheumatol Adv Pract. 2025 Dec 3;10(1):rkaf133. doi: 10.1093/rap/rkaf133 (PMC12758116; doi:10.1093/rap/rkaf133)
Supplement: rkaf133_Supplementary_Data [file rkaf133_supplementary_data.docx]

**Supplementary Table S1.** List of markers used for surface and intracellular staining in CyTOF.

Surface markers

| Antibody | Clone | Metal | Source |
| --- | --- | --- | --- |
| CD66b | 6H6 | 143Nd | BioLegend |
| HLA-DR | L243 | 110Cd | BioLegend |
| CD19 | HIB19 | 111Cd | BioLegend |
| CD8a | RPA-T8 | 112Cd | BioLegend |
| CD16 | 3G8 | 113Cd | BioLegend |
| CD3 | UCHT1 | 114Cd | BioLegend |
| CD4 | RPA-T4 | 116Cd | BioLegend |
| P2RX7 | 7G1D6 | 141Pr | Novus Biologicals |
| CD11c | 3.9 | 142Nd | BioLegend |
| CD123 (IL-3R) | 6H6 | 143Nd | Standard BioTools |
| CD163 | GHI/61 | 145Nd | Standard BioTools |
| CD64 | 10.1 | 146Nd | Standard BioTools |
| CD182 | 5E8/CXCR2 | 147Sm | Standard BioTools |
| CD56 (NCAM) | NCAM16.2 | 149Sm | Standard BioTools |
| CD126 (IL-6R) | UV4 | 150Nd | BioLegend |
| CD107a (LAMP1) | H4A3 | 151Eu | Standard BioTools |
| CD62L (L-selectin) | DREG-56 | 153Eu | Standard BioTools |
| MerTK | 590H11G1E3 | 155Gd | BioLegend |
| CD86 | IT2.2 | 156Gd | Standard BioTools |
| CD284 (TLR4) | HTA125 | 158Gd | Standard BioTools |
| CD14 | M5E2 | 160Gd | Standard BioTools |
| CD319 (SLAMF7) | 162.1 | 162Dy | BioLegend |
| CD206 (MMR) | 15-2 | 168Er | Standard BioTools |
| CD285 (TLR5) | S16021I | 170Er | BioLegend |
| CD226 | DX11 | 171Yb | Standard BioTools |
| CX3CR1 | 2A9-1 | 172Yb | BioLegend |
| CD184 (CXCR4) | 12G5 | 175Lu | Standard BioTools |
| CD282 (TLR2) | TL2.1 | 176Yb | Standard BioTools |
| CD11b (Mac-1) | ICRF44 | 209Bi | Standard BioTools |
| CD45 | HI30 | 89Y | Standard BioTools |

Intracellular markers

| Antibody | Clone | Metal | Source |
| --- | --- | --- | --- |
| IL-18 | 925008 | 144Nd | R&D |
| TNFα | Mab11 | 152Sm | Standard BioTools |
| IL-6 | MQ2-13A5 | 154Sm | Standard BioTools |
| IFNγ | B27 | 165Ho | Standard BioTools |
| IL-10 | JES3-9D7 | 166Er | Standard BioTools |
| IL-1β | 8516 | 169Tm | BioLegend |

**Supplementary Figure S1.** t-SNE analysis of cell surface and intracellular marker expression in a representative case.


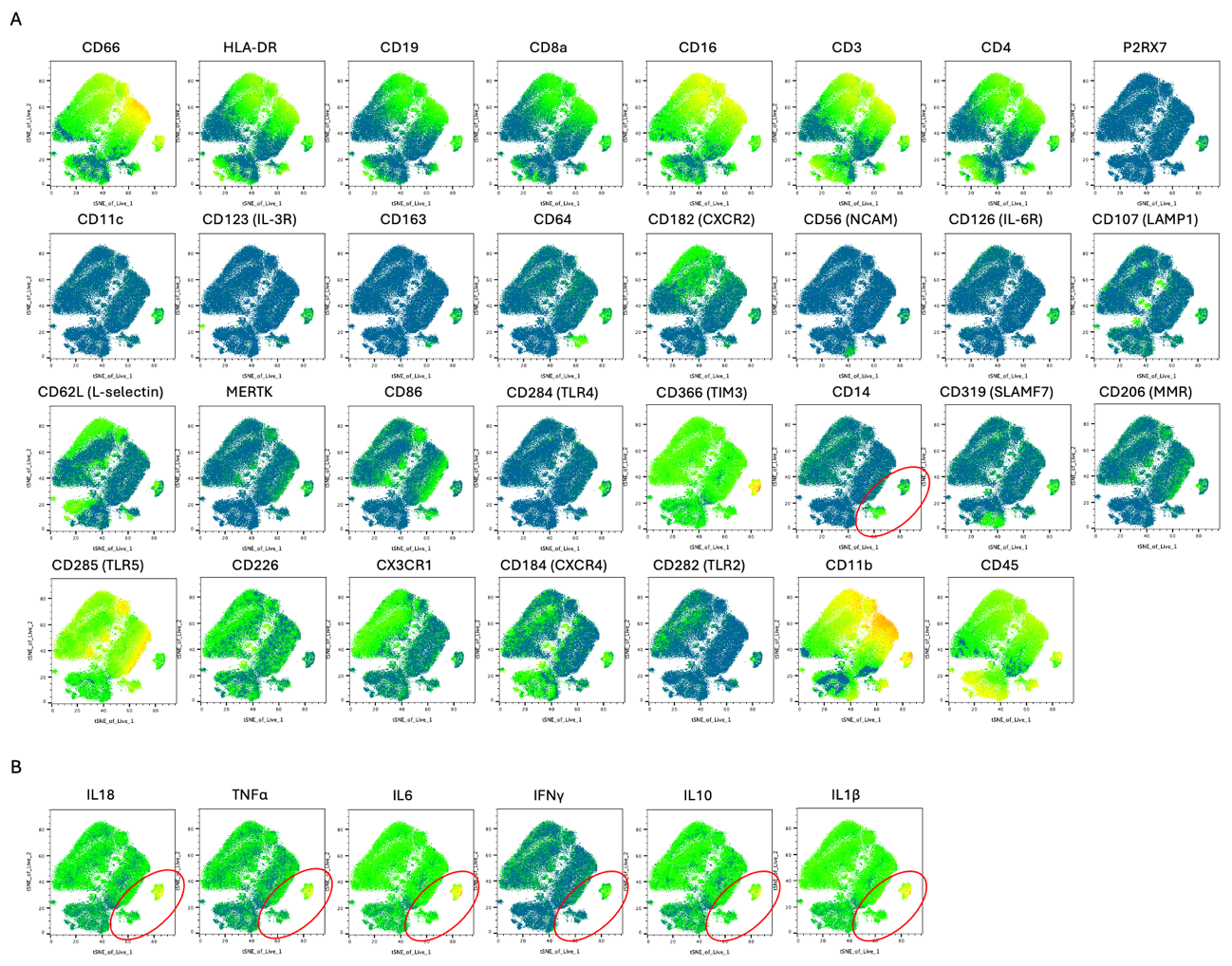


(A) t-SNE plot showing the mapping of cell surface marker expression. (B) t-SNE plot showing the mapping of intracellular marker expression.
